# Agency and Bandura’s Model of Triadic Reciprocal Causation: An Exploratory Mobility Study Among Metrorail Commuters in the Western Cape, South Africa

**DOI:** 10.3389/fpsyg.2019.00411

**Published:** 2019-03-15

**Authors:** Zinette Bergman, Manfred Max Bergman, Andrew Thatcher

**Affiliations:** ^1^ Department of Psychology, University of the Witwatersrand, Johannesburg, South Africa; ^2^ Social Research and Methodology Group, University of Basel, Basel, Switzerland

**Keywords:** sustainable mobility, Albert Bandura, agency, triadic reciprocal causation, mixed methods, hermeneutic content analysis, content configuration analysis, Metrorail

## Abstract

Most studies on sustainable mobility focus on technological, socio-structural, or psychosocial influences while neglecting individual motivations and practices. In this study, we examine mobility motivations and practices as part of a complex interplay between psychosocial and socio-structural dimensions within the mobility infrastructure of Metrorail in the Western Cape. Drawing on Albert Bandura’s theory of personal agency and the model of triadic reciprocal causation, we interviewed 38 commuters (mean age 33 years, SD 11, 50% women/men) and analyzed the data using hermeneutic content analysis and multidimensional scaling. Based on our analyses, we identified three pathways that describe the mobility practices of Metrorail users, each with its own purpose and function. We explore these pathways and their consequences for sustainable mobility in relation to daily commuter agency, motivations, and past experiences.

## Introduction

Despite decades of innovations and interventions, the transport sector still accounts for approximately one-sixth of greenhouse gas (GHG) and CO_2_ emissions (IPCC, 2007, 2014). Consequently, mitigating environmental, health, and social risks caused by mobility practices remain a crucial challenge (Bandura, 2008; Geerken et al., 2009; Guadagno, 2016; Boas, 2017; Yamamoto et al., 2018). More sustainable mobility practices would mean “to reduce the need to travel (less trips), to encourage modal shift, to reduce trip lengths and to encourage greater efficiency in the transport system” (Banister, 2008, p. 75). Such solutions tend to focus on either technical and socio-structural changes or psychosocial interventions.

Technical and socio-structural approaches aim to mitigate GHGs by increasing the efficiency of transport systems. Known as hard policy approaches, they seek to remodel transportation systems through technological and infrastructure development (Novaco, 2001; Brög et al., 2004; Hunecke et al., 2007; Gehlert et al., 2013). Current green technology solutions include mass public transit, high-speed rail, shared and autonomous mobility systems, and electric vehicles. Hard sustainable mobility policies tend to assume availability and affordability of raw materials, industrial capacity, and extensive investment for the development, implementation, and maintenance of disruptive technologies. Also assumed is that, for example through incentives or taxes, the public could be enticed to support the necessary economic, political, and cultural changes that are part of the disruptive technology.

Psychosocial approaches focus on individuals or groups to improve the sustainability of mobility (Brög et al., 2004; Stanton et al., 2013). At the center of the so-called soft approach are individual or collective attitudes, values, norms, motivations, preferences, habits, and behaviors with the goal of creating modal shifts in why and how individuals or groups travel (Novaco, 2001; Steg and Vlek, 2009; Gehlert et al., 2013). Public appeals and awareness campaigns are currently the main tool to increase knowledge and acceptance of sustainable mobility (Brög et al., 2004; Hunecke et al., 2007). Included under this rubric are approaches that focus on inequality (Titheridge et al., 2014; Zhao and Li, 2016), inclusiveness (Bergman et al., 2014; OECD, 2016), access (Starkey and Hine, 2014; Bergman and Bergman, 2015; World Bank, 2016), and ecological behavior (Collado et al., 2013; Lokhorst et al., 2013; Pillemer et al., 2017; Landry et al., 2018). Compared to hard policy measures, psychosocial interventions tend to enjoy a greater degree of political support because they can be implemented at significantly lower cost and with fewer systemic disruptions (Stanton et al., 2013). However, psychosocial interventions encouraging modal shifts have had limited success partly because initiatives and policies tend to emphasize *how* people travel, not *why* they travel (Cass and Faulconbridge, 2016), and because they often neglect environmental constraints, such as shortcomings in mobility infrastructure or other structural barriers (Bergman et al., 2014; Bergman and Bergman, 2015).

With few exceptions (e.g. van Wee et al., 2002; Poortinga et al., 2004; Collins and Chambers, 2005; Hunecke et al., 2007; Steg et al., 2014), most mobility studies focus on either technical and socio-structural or psychosocial dimensions of mobility. Hunecke et al. (2007), for example, examined the effect of psychological, socio-demographic, and infrastructure influences on the ecological impact of mobility behavior. Other notable studies include Poortinga et al. (2004), who analyzed the impact of value dimensions concerning different psychological and environmental aspects on home and transport energy usage, and Steg et al. (2014), who studied the impact of values, situational cues, and goals to encourage pro-environmental behavior. Finally, van Wee et al. (2002), Collins and Chambers (2005) and Hunecke et al. (2007), explored psychological, sociodemographic, infrastructural, or situational effects on mobility behavior. Despite diverse foci, these studies arrived at similar conclusions: mobility practices are nested within a complex network of individual, social, and environmental factors, and the consensus seems to be that we need to better understand the synergy between individuals and their mobility environment in order to exploit the potential of behavior change toward more sustainable mobility (Shepherd and Marshall, 2005). Given the relative neglect of the interdependence between commuter motivations and practices in specific mobility environments, little is known about what a sustainable transportation system would look like, what criteria should be used to assess it, or what would make it socially and culturally acceptable (Steg and Gifford, 2005). Charlton (2004, p. 165) argues that “[u]nless these complex, interrelated socio-economic and behavioral influences can be properly interpreted and, crucially, incorporated into policy and practice, genuine advances to greater sustainability will be elusive.”

In this article, we first present Albert Bandura’s triadic reciprocal causation as a suitable theoretical framework that can account for the complex interdependence among mobility intentions, practices, and the environment in which they are embedded. We then present an empirical application of this framework to analyze mobility practices of train commuters in the Western Cape, South Africa. The overall aim of this article is to contribute to a better understanding of individual agency within specific mobility environments in order to improve conceptualizations and implementations of sustainable mobility solutions.

## Theoretical Background

We encounter multiple and constantly changing environments each day, requiring a vast array of choices. Despite ever-changing dynamics, we manage to negotiate a highly complex world because our behaviors are neither hardwired nor mere products of our environment. As active agents, we influence outcomes, we act upon others’ behavior, and we coordinate behaviors with each other (Bandura, 2006).

*Social cognitive theory* (SCT) as developed by Albert Bandura proposes that human behavior encompasses core features that include not only internal behavioral predispositions, such as cognition, affect, or motivation, but also various environmental influences (Bandura, 2001). SCT terms deliberative behavior *personal agency*, which has been studied extensively in psychology (Bandura, 2006, 2008), public health (Bandura, 2004a), education (Rogers et al., 1999; Chapman-Novakofski and Karduck, 2005), business and management (Schmutzler et al., 2018; St-Jean et al., 2018), and media studies (Bandura, 2004b; Gibson, 2004). Personal agency refers to an individual’s ability to “designedly conceive unique events and different novel courses of action [while choosing] to execute one of them” (Bandura, 2001, p. 5). It includes complex processes of intra-personal cognitive processing, deliberation, and decision-making, motivated by a desire to achieve specific outcomes. According to Bandura, desires shape our *intentions* to act, thus preceding behavior toward goals or aspirational ends. Subsequently, desires to achieve an end serve as the impetus for, and the intended outcome of, our actions. The process of turning intentions into *goals* involves a number of decision-making strategies. The first relates to three modes of agency: *individual*, *proxy*, and *collective*. According to Bandura (2001), individual agency entails the process whereby people deliberately guide their behavior within an immediate environment. If the goal is to get to work, for example, we may elect to drive our car or ride bicycles. Individual agency has its limits because individuals may not always be able to act on their own behalf. Children, for example, are unable to drive cars, and they may not own or be allowed to ride their bicycle to school. In this case, agency without the assistance of others is impeded. Proxy agency involves enlisting others to act on our behalf to secure desired outcomes. Collective agency refers to collective efforts to achieve a desired outcome through interdependence and the activation of networks (Bandura, 2008). This might entail organizing a car sharing club or petitioning local politicians to fund a public transit system. Each mode offers a different way to achieve a goal, and despite cross-cultural variations, we rely on all three modes of agency to conduct our lives (Bandura, 2001).

Agency is mediated by contextual and cultural influences, such as “activities, situational circumstances, and socio-structural constraints, and opportunities” (Bandura, 1999, p. 6). It is preceded by an assessment of opportunities and constraints inherent in socio-structural or contextual environments (Bandura, 2001). Environments are assessed and perceived to facilitate or hinder the ability to act. Car ownership and lack of access to public transport are examples of components of mobility environments that facilitate or obstruct mobility pathways. Bandura (2001) distinguished between three environments, namely the *selected*, the *constructed*, and the *imposed* environment. The selected environment provides the largest scope of behavior and therewith the broadest agentive space. Here, individuals are agents of their realities, they have at their disposal a range of different behavioral options, and they can choose behaviors that best suit a desired outcome in a specific situation. By choosing “associates, activities, and milieus,” environments are selectively activated as individuals formulate appropriate courses of action and decide how to behave (Bandura, 1999, p. 6). In terms of mobility practices, a selected environment may include access to mobility modes, such as a car, bus, or train. The modal choice reflects whatever is perceived to best achieve a desired outcome. The constructed environment requires concerted effort to become a viable agentive option. It restricts agentive practice because it requires “people to construct social environments and institutional systems through their generative efforts” (Bandura, 1999, p. 6). Examples include arranging a ride in a car sharing club to get to work, campaigning for public transport systems to be extended into a township, or relocating to reduce the distance to a train station. The imposed physical and socio-structural environment narrows the scope of agency because it dictates the boundaries within which people behave and, although “they have little control over its presence, they have leeway in how they construe it and react to it” (Bandura, 1999, p. 6). For example, walking long distances to school as the only form of available mobility reflects an imposed environment. Agency still exists in which the pupil may choose whether to attend school on a given day, or which route to take to avoid anticipated hazards.

**Figure 1:**
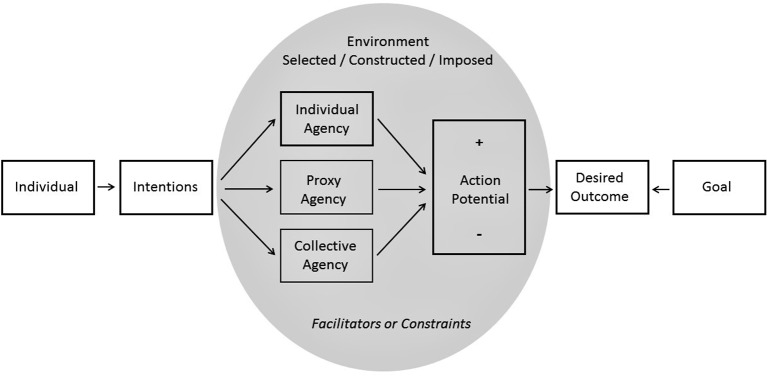
Model of Bandura’s personal agency and triadic reciprocal causation.

The modes of agency and their environments are interdependent. According to Bandura (2006, p. 6), “internal personal factors in the form of cognitive, affective, and biological events, behavioral patterns, and environmental influences all operate as interacting determinants that influence one another.” Derived from SCT, Bandura’s model of triadic reciprocal causation (Bandura, 1986; see also Bandura, 1989, 1999, 2001, 2006) emphasizes that personal agency is inherently psychosocial and functionally dependent on events. Accordingly, agency may be presented as follows:

Figure 1 models individuals’ intentions to achieve desired outcomes. Through complex processes of intra-personal deliberation, individuals assess how various environments (selected, constructed, or imposed) facilitate or constrain their potential to act (action potential), as well as how different modes of agency (individual, proxy, or collective) enable them to achieve their goal. Based on deliberations within environments, individuals choose the mode of agency (individual, proxy, or collective) that will most likely secure a desired outcome in a specific context. An appropriate course of action is then selected and implemented as people adjust their behavior accordingly.

Given the variability of options and conditions, it follows that no fixed, predictable pattern of reciprocal interaction exists (Bandura, 2006). The uniqueness of the constellation of agency in a given environment makes agency inherently difficult to study. One of the main criticisms aimed at Bandura’s work relates to the relative looseness of the concepts and their interdependence (Tschannen-Moran and Hoy, 2001; Garvis and Pendergast, 2016). Others argued that the conceptual overlap between expectations (intentions) and outcomes limits the applicability of the theory (Eastman and Marzillier, 1984). Consequently, most SCT studies reduced agency to individual agency, focusing predominantly on self-efficacy to model behavior change, such as in the field of health, especially therapeutic research (Bandura, 1997; Langlois et al., 1999), preventative health (Tougas et al., 2015), public health education (Ryerson, 1994; Rogers et al., 1999; Bandura, 2004a; Chapman-Novakofski and Karduck, 2005), education (Bores-Rangel et al., 1990; Church et al., 1992; Hackett and Byars, 1996), and media studies (Gibson, 2004; Hill et al., 2009). The selective focus on intra-personal, cognitive dimensions of individual agency limits accounts of human behavior in situations that transcend the confines of unidirectional modes of causation (Bandura, 1999). The fact that most studies have applied only one type of agency from SCT (Carillo, 2010) and the consequences of this one-sided application represents the most compelling critique against studies on personal agency.

We seek to expand the conventional, unidirectional application of SCT by exploring the multidimensional nature of personal agency as initially formulated by Bandura and by applying triadic reciprocal causation to mobility practices of Metrorail commuters in the Western Cape. The rationale for this study are three-fold. First, we aim to study human agency using the model of triadic reciprocal causation to expand the applicability of Bandura’s theory. This means situating individual, proxy, and collective components of agentive practice within psychosocial and socio-structural environments. Second, we apply this multidimensional concept of agency to study mobility practices. Specifically, we propose to analyze agency and mobility practices in the context of Metrorail commuters in the Western Cape, South Africa. Metrorail is the largest commuter train service in South Africa, transporting approximately 2 million people every day on 2228 km of track. The local Metrorail network in the Western Cape region has been operational since 1863. It consists of four main lines – a Northern Line, Southern Line, Cape Flats Line, and Central Line with 610 km of track and 119 stations that connect informal settlements, townships, suburbs, towns, and cities in the South Western Cape. Third, by exploring mobility with Bandura’s multidimensional approach to agency, we hope to contribute to a debate on sustainable mobility that goes beyond interventions, which focus on either technical and socio-structural or psychosocial manipulations. In other words, Bandura’s theory of agency will be used in a case study to argue for a reciprocal relationship between technical, socio-structural, and psychosocial effects on mobility behavior. Our rationale translate into three research questions:

1. Can we empirically identify the agency and environment dimensions outlined in Bandura’s model of triadic reciprocal causation in the narratives of Metrorail commuters?
2. How do the dimensions of agency and environment interrelate in the reported mobility practices of Metrorail commuters?
3. What are the implications of conceptualizing agency accordingly on the understanding of sustainable mobility systems for Metrorail commuters?

## Materials and Methods

### Sample

This study is based on 38 narrative interviews with Metrorail commuters in the Western Cape. Three selection criteria assisted in identifying eligible participants: mobility type (use of Metrorail), frequency (week-day commutes during that past 2 years), and geographical location (multiple commutes per week in the wider Cape Town or Stellenbosch region). The interviewed men (*n* = 19) and women (age range 18–62 years, mean 33 years, SD 11) were multi-ethnic (black, white, and colored^1^), multi-lingual (speaking predominantly Afrikaans, isiXhosa, and English, as well as Tswana, Sesotho, Sotho, and isiZulu), and pursued a variety of occupations (students, teachers, security guards, shop attendants, cleaners, drivers, administrators, couriers, repair men and women, managers, occupational therapists, personal assistants, and unemployed). All recruited participants took part in the interview. The coding of the interviews yielded 784 codes for the multidimensional scaling (MDS) analysis, which is regarded as an adequate sample size for a dimensional analysis (de Winter et al., 2009). Considering the small size of the sample, we could not control for the effect of individual differences.

### Procedure

Before commencing data collection, we obtained permission to conduct the research from the University of the Witwatersrand Human Research Ethics Committee. Based on our sampling criteria, participants were recruited near train stations in Cape Town and Stellenbosch. Interviews were conducted immediately, or at an arranged time near the station (in public spaces or cafes), or at a venue negotiated between the interviewer and the participant. All requests for interviews were accepted. Interviews were conducted in English or Afrikaans (the two dominant languages in this region). The interviews averaged approximately 40 min. All interviews were recorded, transcribed, and anonymized for analysis.

### Instrument

The interview schedule was developed and refined during two pilot phases with members from the research population. The interview schedule included exploratory and semi-structured questions. Initially, exploratory questions aimed to elicit extended narrative responses from interviewees regarding their mobility experiences. Question included “Tell me everything that comes to mind when you think about trains” or “What is your best memory with a train?” These were followed by semi-structured questions aimed to prompt specific mobility preferences or to examine mobility dimensions in detail, such as “When, where, and how often do you take trains?” or “What do you think will happen with trains in the future?”

### Analysis

Data were analyzed using hermeneutic content analysis (HCA; Bergman, 2010), a three-step mixed methods approach. First, interviews were analyzed using content configuration analysis (CCA; Bergman, 2011; Bergman and Bergman, submitted). CCA is a qualitative method used for the systematic analysis of non-numeric data, closely related to qualitative content and thematic analyses (Bergman et al., 2011). For this article, interview data were coded top-down, using the dimensions of personal agency as proposed by Bandura. Due to the mobility focus of our study, we also coded intentions and outcomes that pertained to mobility within mobility environments. The coding scheme was developed and applied in a research team. Two independent coders applied the coding scheme iteratively until the emergent coding taxonomy stabilized. The purpose of the initial CCA was to trace dimensions of agency and environment as outlined by Bandura in the context of Metrorail commuters in the Western Cape. In the second analytic step, we identified agentive pathways and mobility environments, using MDS, which enabled a geometric representation of co-occurrences between agency and environment dimensions. We calculated similarity matrices using the Jaccard Index based on thectar (Berger, forthcoming) and smacof (Mair et al., 2015) in R. The unit of comparison was at code-level (*n* = 784), and the parameters included a non-metric procedure with a primary approach to ties. Stress was at 0.11, which is considerably lower than the stress level for a random sample of the same number of points in MDS, estimated at 0.24 (Spence, 1979). A two-dimensional map was found to be the most parsimonious and interpretable solution. Adding an additional dimension did not significantly improve stress but worsened interpretability and parsimony. The third and final step of HCA consisted of a re-contextualizing qualitative analysis to connect the MDS structures to the interview data, again using CCA. This step helped interpret the meaning of the MDS patterns by referring back to the interview data in which the MDS structures were embedded.

## Results

In our analyses, we identified the intra- and inter-personal, as well as socio-structural environmental dimensions that delineated mobility practices, explored the relations between dimensions of agency, and conceptualized “mobility as agency” from the perspectives of Metrorail users.

### Dimensions of Personal Agency

In the first step, we coded and analyzed the interview data deductively to explore Bandura’s tripartite agency concept. This entailed identifying intentions, types of agency, environmental facilitators and constraints, action potentials, and desired mobility outcomes. The following example illustrates this analytic step:

I just want to walk down there this afternoon and hope the train will be on time. … No, honestly, no man, it’s a headache I tell you. No, I don’t even want to think about it. Because you see actually I leave here at 17:15, right? The train is actually supposed to be there at half past 5, but there is no way that I will waste my time and walk quickly because I know it will either be late or it would have left already. Do you see? So then I rather take the 6 o’clock train. Even if that means I only get there by 18:30. On the other side, the whole fact of the matter is that you need to get home. It doesn’t matter what time you leave here, you simply need to get home. How you are going to get there, what time you will get there, that is simply your own damn problem. And it shouldn’t be like that. It really shouldn’t be like that. (MT3: 3)

#### Intentions and Desired Outcomes

In this excerpt, agency in relation to mobility began with intentions to be mobile. According to Bandura, intentions consist of the intra-personal cognitive processing of personal needs or desires in relation to anticipated contextual factors and potential desired outcomes. The intention “to get home” connected to what the interviewee perceived as the most significant contextual issue, the unreliability of the train, because it had implications on when he would get home. This assessment enabled him to identify the best course of action – to take a later train. The overarching goal here was to align his intentions with perceived outcomes. Bandura proposed that desired outcomes relate to the extent to which intentions may be realized. Deliberation and making decisions based on when to leave work, when to reach the station, and which train to take assisted this commuter in achieving his goal or desired outcome.

#### The Environment as a Facilitator or Constraint

The ability to be mobile is mediated by environmental factors. They dictate mobility boundaries and enable or prevent agentive practices. The excerpt above exemplified a late train as a contextual factor that represented an obstacle to the interviewee’s ability to be mobile. This situational circumstance illustrates facilitating or impeding environmental factors mentioned by most Metrorail users in this study. Table 1 below summarizes the environmental facilitators and constraints from our interviews.

**Table 1:** Examples of environmental factors mentioned by Metrorail users.

Environmental facilitator	Environmental constraints
Safety	Lack of safety
Comfort	Overcrowding
Cleanliness	Dilapidated, broken-down, and out-dated infrastructure and train stations
Efficiency	Service disruptions
Reliability	Delays
	Unavailability of Metrorail staff and information

Two characteristics are noteworthy in this table. First, environmental facilitators and constraints lie on opposite ends of a dimension, for example, a train that was on time and a train that was cancelled, respectively. Second, interviewees identified many more constraints than facilitators, a predominant trend in the data since the constraints that restricted agentive practice were far more prevalent not only in frequency but also in terms of perceived significance and degree. This means that our interviewees focused overwhelmingly on experiences associated with constrained mobility environments. The previous and following excerpts illustrate environmental constraints:

I mean we pay, even though we pay less but we pay. There are so many commuters. We buy so many monthlies [monthly tickets]. How much money does Metrorail make? Why can’t they do that? Why can’t they give us a comfortable, convenient environment to sit in? (MT1: 6)

Another noteworthy dimension underlying environmental concerns is that facilitators tended to be aspirational, hypothetical, or future-oriented, such as a planned expansion of train line, in contrast to environmental constraints, which were presented as common experiences, such as unsafe and unreliable trains during rush hour.

#### Selected, Constructed, and Imposed Environments

According to Bandura, an important feature of environmental facilitators and constraints concerns gradation of variability. Contextual dimensions are imposed, constructed, or selected. The train delay from the first excerpt was an example of this: the commuter may have decided to take an earlier or later train – an instance of bounded agency – but he lacked alternative modal choices. He does not own a car and cannot afford alternative modes of transport. Consequently, he lacked the ability to select or construct a different mobility environment. This illustrated the impact of an imposed environment since his environment and access to resources dictated the boundaries of his action potential. In this way, environmental facilitators and constraints impose a range of variability within which individuals can respond. All three environments shape actual and potential mobility options.

#### Individual, Proxy, and Collective Modes of Agency

The modes of agency in the previous excerpts related to perceived abilities to be mobile based on a relational dependence. Bandura termed this proxy agency – when others act on the agent’s behalf. Here another example of proxy agency:

So even now, if the car were to break down, I wouldn’t even take a taxi. I would just call someone to come and fetch me. Like my nephew or someone. I wouldn’t walk or take a taxi, not unless I really have no other choice. But that just goes to show how convenient and comfortable my life has become. (MT9: 1)

The interviewee did not elect walking or using a taxi because she considered these modes inconvenient or unsafe in relation to another option. She constructed an alternative mobility option by enlisting someone else, a proxy agent (“I would just call someone to come and fetch me. Like my nephew or someone.”). In our data, the most frequently mentioned proxy agent was Metrorail. For example, an interviewee wished he could rely on this proxy to act on his behalf [“How you are going to get there, what time you will get there, that is simply your own damn problem. And it shouldn’t be like that. It really shouldn’t be like that.” (MT3: 3)]. Commuters frequently expressed their desire for Metrorail to act as a proxy agent to improve their train experiences, such as requests to increase the frequency and the reliability of trains, to enhance the convenience and comfort of trains, or to improve safety and security.

Individual agency relates to instances where commuters deliberately guided their behavior *via* mobility options at their disposal. Some commuters reported that Metrorail was their only mobility option, while others were able to limit train use to weekday commutes and made use of alternative mobility modes in other life spheres. In some cases, the obstacles commuters encountered resulted in abandoning Metrorail. For most, Metrorail was the least preferred mode of mobility and the first to be replaced, if other modes became available. Here an example:

So, I take [Metrorail] regularly. Yea, yea, yes, I take it Monday to Friday, weekends I don’t bother with the trains at all, like I’ve told you. We prefer to take the vehicle on the weekend of course it is going to work out more expensive but you can do so much more with the vehicle because then you can do your shopping and things like that. You see, because I am actually one of those fortunate ones because those other people have to also do their whole shopping with the trains, right? They are not as fortunate as some of us. But of course it costs a lot of money. (MT3: 6)

Collective agency refers to acts of interdependent effort that enabled individuals *via* groups or a collective to achieve a goal. In the case of our Metrorail commuters, collective agency referred mostly to the activation of social ties, often based on religious, friendship, or work networks that developed during train commutes. Here, an example:

…the positive thing that I learned out of [being unable to afford a car] was that God wanted to place me among people because He knows my heart and He knew that I have a need that burns inside of me to serve Him, and this is why I was short of money. But I have become richer in Him because now I have a social group that I have every day, they can feed me, they can give me provisions for the road, they can comfort me and this is really the thing that stands out the most for me about taking the train because I learn every day and I realize every day and I become wiser every day through them because I take the train. (MT1: 4)

Table 2 summarizes the modes of agency of Metrorail users.

**Table 2:** Summary of the modes of agency mentioned by Metrorail users.

Individual mode of mobility	Collective mode of mobility	Proxy mode of mobility
Metrorail	Religious networks	Metrorail
Buses	Friendship networks	Family members
Taxis	Work-related networks	
Privately owned vehicles	Car/lift sharing arrangements	
Walking		

#### Action Potential

Modes of agency, environmental facilitators, and constraints and the ability of commuters to assess agency according to their environment relative to their capabilities, intentions, and desires contributed to action potential. The action potential manifested positively or negatively, depending on how these dimensions combined. Positively framed, the reciprocal interaction between agentive and environmental dimensions enabled commuters to achieve their mobility goals. Negatively framed, some commuters were unable to overcome obstacles to mobility, based on environmental constraints or a lack of agency. Their action potential was restricted and their mobility desires remained unfilled.

In this analysis, we connected Bandura’s proposed dimensions of agency and environment to the mobility practices of Metrorail commuters and found that all dimensions were present in the narratives of Metrorail commuters. Next, we examined the interdependence these dimensions.

### Systematizing the Interdependence of Dimensions of Personal Agency and Environment in the Reported Mobility Practices of Metrorail Commuters

**Figure 2:**
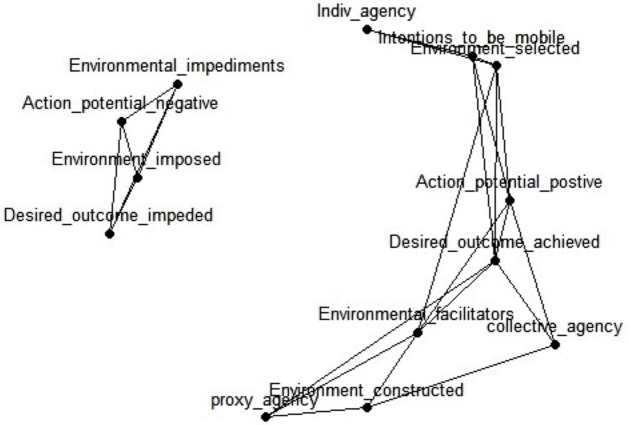
MDS map of the agentive practices of the Western Cape Metrorail commuters.

The narratives on mobility experiences were composed of unique constellations of intentions and goals, facilitating or constraining environmental factors, and modes of agency. While the first set of analyses examined the presence of dimensions as outlined by Bandura’s triadic reciprocal causation model, mapping them systematically deepened our understanding of agency in a specific mobility environment. To do this, we used a dimensional analysis, specifically MDS, to map the relations between Bandura’s agentive dimensions and to visualize mobility structures in an *n*-dimensional space. The representation of relations was facilitated by dividing the dimensions’ action potential and desired outcome into positive or negative constituents – action potential positive and action potential negative, and desired outcome achieved and desired outcome impeded. Mapping patterns of agency in a specific mobility environment revealed distinct patterns of reciprocal interaction between agentive practices and environments. We present this in Figure 2.

Represented in Figure 2 are the dimensions of agency and environment as points in a two-dimensional space. The distance between points represents the relative frequency or co-occurrence of dimensions in the interview data. The closer the points are located to each other, the more frequently the dimensions co-occurred. Conversely, the further apart these are, the fewer the co-occurrences, and the more orthogonal the dimensions are to each other. Consulting the interview transcripts assisted the interpretation of this map.

According to Figure 2, the agentive practices of Metrorail commuters are divided into two main clusters: a small cluster on the left, which we will refer to as cluster 1, and a larger cluster on the right, cluster 2. Linking the top 30% of co-occurrences with a straight line visualizes the two-cluster structure. The second notable feature relates to the shape of the clusters. The dimensions of cluster 1 are situated in close proximity to one another. Each component connects to all others in this cluster. Except for one dyad, the points in this cluster are roughly equidistant to each other. The elongated, crescent shape of cluster 2 on the right indicates that the dimensions in this cluster are connected. In contrast to cluster 1 there are some interpretable differences in this cluster, given the relative distance between the dimensions situated at the top and bottom of the crescent. Finally, the elongated shape of cluster 2 is approximately equidistant to cluster 1. Five notable findings can be inferred from this map.

First, cluster 1 includes four dimensions: environmental impediments, a negative action potential, an imposed environment, and the impediment of a desired outcome. This is interesting because the cluster contains all dimensions, which restrict agency (imposed, impeded, and negative). These dimensions are highly interdependent, given not only the geometric proximity of the points but also their connectedness. This cluster is geometrically and thus conceptually different from the other agency and environment dimensions. Based on the content and location of this cluster, we observed that the challenges and obstacles that impeded mobility agency and practices of the Metrorail users were intertwined. These included references to their imposed mobility environment, such as dilapidated infrastructure or inadequate services, as well as environmental impediments, such as delays and breakdowns in the system. The consequences connected to these obstacles were negated agentive practices and inhibited desired outcomes as they resulted in restricted agentive practice. Given the relative distance to personal, proxy, or collective agency, this cluster represents the opposite of agentive practice – the lack of agency. The following are two excerpts to illustrate the nature of cluster 1:

There were some days, sometimes when people have stolen the power cables. So then, people can’t go to work for at least a day or will be late by two or three hours. So yes, I think this is actually a terrible experience, especially if there is work to be done. They steal the cables a lot, yes. (MT2: 2)

For example, yesterday morning. My train is at 5:45AM. No announcements, nothing. The train arrives at 6:30AM. Do you see? Now I have to let the people at work know that the train is late, but they don’t understand. It’s very frustrating. (MT3: 1)

Second, individual agency is located at the top point of the crescent in cluster 2. It connects to intentions to be mobile and the selected environment. Both dimensions are connected to a positive action potential and achieving a desired outcome, while a selected environment is further connected to a facilitating environment. Three characteristics can be observed in this part of the figure: 1) Individual agency seems to be a cornerstone of a network of connections between mobility dimensions. This part of the constellation implies that individual agency involves a number of core features including intentions to be mobile and the ability to select an environment that facilitates the action potential of the individual to achieve a desired outcome. 2) The dimensions of this part of the cluster are active and positive manifestations of agency. 3) This agentive pathway is similar to the classical understanding of personal agency, where individuals (in our case, commuters) are full agents in the sense that they have different (mobility) options. Here an illustrative excerpt:

I mean, it’s so many commuters, I mean, me coming from the sustainable side, that’s what I’m all about that, I’m all about green living. I prefer using public transport. I have a vehicle but coming to work, I use public transport. Even if it means a taxi or bus, I use it because, I mean, more people in one vehicle, automatically we’ll be saving the environment slowly. (MT24: 1)

For this commuter, individual agency shaped mobility. It transcended commuting from point A to B based on imposed options. Instead, it included an overarching goal of living a greener, more sustainable life, resulting in a concerted effort to utilize mobility options that made this a viable agentive option. Accordingly, the commuter adapted her mobility choices and selected an environment that best suited her desired outcome.

Third, proxy agency is located at the lowest point of cluster 2, which is closely associated with a constructed environment. Proxy agency is also connected to environmental facilitators and achieving a desired outcome. Similar to the constellation located at the top, we identified an agentive pathway at the bottom of this cluster. In this part of the constellation, proxy agency is associated with the ability to construct an environment on behalf of commuters. Interestingly, this agentive pathway is not directly connected to the action potential of commuters, a point we will return to later. The examples mentioned previously that related to Metrorail acting on behalf of commuters, or reaching out to friends and families during emergencies, were indicative of this agentive pathway. Here another example:

Stick to time, and send out notices, like if they know people are using trains regularly, like if there’s a delay, send SMS’s to people, you know, be like “the trains will be delayed like an hour,” like give me a choice, help me decide if I want to take the train or a taxi, maybe I could’ve compromised or made another option, but now I don’t know, I get there, now I wait, and it’s like five minutes, then you wait, then it’s like forty minutes, you know, so yeah. Like, let people know. (MT32: 3)

Fourth, the final type of agency in Figure 2, collective agency, is located near the center of cluster 2. It is connected to three agentive dimensions, namely the construction of an environment, a positive action potential, and achieving one’s desired outcome. This constellation implies that collective agency consists of a co-construction of mobility environments through interdependent efforts, which increase the potential for agency. This agentive pathway is interesting for several reasons. In contrast to individual and proxy agency, which are situated at opposite ends of this cluster, collective agency is located centrally, close to achieving one’s desired outcome. The proximity of these two dimensions indicates that this agentive pathway is closest to Metrorail users achieving their desired outcomes. Nearly equidistant from proxy agency and individual agency, it also implies that it shares some characteristics with these. It appears that collective agency is made up of individual and proxy agency while being more effective than the single individual or the proxy agent.

Fifth, and as a consequence of the above, it is more appropriate to think of this agency cluster not as a crescent but rather as a continuum, where collective agency represents the mid-point between individual and proxy agency. One way to make sense of this is to consider the locality of agency along this continuum. At the top, Metrorail commuters are directly involved as individual agents in determining their mobility outcomes. Here, agency resides in the individual and represents deliberate personal action (the “I” and “me”). At the bottom, people are only indirectly influencing the outcome as they rely on someone else to act on their behalf. Here, agency and the ability to achieve a desired outcome reside with a proxy (“they” and “the others”). In the middle, agency is shared through interdependent effort as part of the collective agency pathway (“we” and “us”). The reason for this elongated cluster to bend into a crescent around the smaller cluster 1 is that all three interconnected agencies are different from, and thus maximally distant to, the non-agentive cluster 1. Finally, despite their commonalities, the modes of agency are relatively distant from each other, which supports Bandura’s argument that individual, proxy, and collective agency are different forms of agency. This difference is due in part to the distinct patterns of reciprocal interaction between the psychosocial and socio-structural environmental dimensions of agency. The illustration of this difference in agency types and their differential relation to environments was one of the goals of this analysis. According to the MDS results, we found that mobility as agency from the perspectives of Metrorail users consisted of three distinct agentive pathways, which were differentiated not only in terms of the locality of agency but also in how they related systematically to different mobility environments.

### Mobility as Agency: The Agentive Pathways of Metrorail Commuters in the Western Cape

In this analytic step, we re-contextualized key patterns in the MDS map according to HCA (Bergman, 2010), which allowed us to better understand the meaning of the MDS patterns as described above.

#### The Individual Agentive Pathway

How did Metrorail commuters achieve individual agency, considering the many challenges inherent in the mobility system? Re-contextualization revealed that commuters primarily used the individual agentive pathway to overcome or avoid Metrorail’s weaknesses, which included alternative modal choices to overcome delays or breakdowns, such as borrowing or buying a car, or using buses or taxis, where available. Here are some examples:

Um, people get into trouble at work because [the trains] are always late. Often I can go back and fetch my car and go with my car but many thousands of people don’t have a car that they could take. This is their only transport. I use it because it is cheaper and because I can read while on the train. I can’t read while I am driving. (MT5: 2)

It’s ridiculous. I mean I used to use the railway but you cannot get to work late. I mean half-an-hour late, more than three times within a month and blame it on public transport. I mean after a while, I’m very sure, that your employer thinks it’s your fault. (MT24: 1)

*Interviewer*: Why don’t you take trains more often?

*Interviewee*: Really? No way, no, no. The thing is, like, if there is a possibility, I would not take a train, if I had a substitute. I would rather take the substitute. But the thing is, this is the cheapest form of transport. But preferably I would rather go by the car. Unfortunately, which I don’t have, but that’s not, no. (MT20: 7)

An interesting variant of this agentive pathway related to commuters exploiting systemic weaknesses to achieve desired outcomes, as illustrated by this excerpt:

Cause if, say tomorrow, a better service than the trains were to come at an affordable price, I promise you, people would stop using the train. It’s just that it’s affordable and it’s easier to use a train, when you don’t even have money or a ticket for some people, because I know guys who live in my street and they wake up early and they leave at four to go to the train, they catch the train for free and then they come back after eight – there’s no guards or anything. So, they don’t buy tickets; they just ride for free. (MT15: 7)

Getting up very early and coming back late at night, or returning home to fetch one’s car when a train is cancelled are examples of how individuals used selected environments to adapt to challenging situations to create viable agentive options. However, such individual agentive practices were exceptional because only a small number of commuters in our sample had access to a car, or were able to commute early or late enough to avoid certain environmental obstacles. Most commuters traveled during peak hours and reported feeling trapped in a deficient mobility environment, since they lacked the means and access to viable alternatives. The majority of mentions connected to this pathway were related to expressions of preferences, wishes, and aspirations; they referred to what commuters wished they had.

*Interviewee*: I don’t know, if you know the movie “The Italian Job?”

*Interviewer*: Yes, yes…

*Interviewee*: Did you see that scene where they’re sitting in that train that looks like a spacious, expensive one. Wouldn’t you wish to be there, like to use that train as a form of transportation?

*Interviewer*: Yeah, of course. But that’s in the movies.

*Interviewee*: Yeah, that’s what I’m saying. You only see them on TV and you wish, why don’t we have that, you know? If they’re coming up with something new that you’d wish, that you’d use as a substitution for your trains. It’s either not around you, or it’s too expensive for you. For instance, the Gautrain, that’s a nice train, you know? But where is it? It’s only in Gauteng, not around South Africa. And certain people use it, not everybody is using it, you know.

*Interviewer:* What do you mean with certain people?

*Interviewee:* People of that region. People who can afford it. Because even there, not everybody is using it. It’s people who can afford it, because maybe the prices are high, I don’t know, but why do they not make it for all of us. Not like the trains that we’re using now. (MT25: 9)

The re-contextualization revealed the limitations of the individual agentive pathway. The ability to choose from a variety of mobility options that facilitate agentive goals tended to be unrelated to daily commuting experiences. Instead, this agentive pathway was predominantly aspirational, confined to wishes or outcomes that manifested in an imagined present or distant future.

#### The Proxy Agentive Pathway

In the proxy pathway, commuters often looked to other agents to act on their behalf. The initial analysis revealed that proxies included, most prominently, Metrorail, but also close family members and friends, lift clubs, and members of personal networks. A re-contextualizing analysis showed that common to all proxies was an ability to construct a new or different mobility environment for commuters. This was obvious when considering the facilitating effect of a lift club, a ride with a work colleague to or from work, or being rescued when stranded. In relation to Metrorail, however, the agentive pathway was obfuscated. While expectations toward the mobility environment were often clearly communicated, facilitating environments rarely materialized as commuters reported that Metrorail was either unwilling or unable to intervene on their behalf. Here are some examples:

*Interviewee*: Um, I think it will just become more and more neglected. The whole train network will just become more and more neglected.

*Interviewer*: Why do you say that?

*Interviewee*: Because all the signs are there that there is no focus on maintenance. I don’t think that Transnet [the State holding company of Metrorail] or Metrorail or whoever has the ability to do it and I also think that they don’t have the money to maintain everything or to keep it at an acceptable level. So that’s the picture that I see – a negative picture… (MT21: 3)

So they really need to implement something of that kind to improve their service. They really need to, they really need to improve their service. Funny enough, I actually saw the other day in the Argus [local newspaper] that they are planning to, but you know they always make plans and make plans and make plans and nothing ever comes from it. The Minister of Transport has just the other day, there was an article in one of the newspapers, they are planning to do something but they never get so far as to actually deliver anything. So, it’s really, it’s really a problem you know. It is a big problem and unfortunately this is the way it is… (MT3: 3)

*Interviewer*: So, what do you think will happen in the future?

*Interviewee*: In this current state? Nothing. If nothing happened for two decades, what will change now and in the future? I believe it was two years ago you know our president? They bought new trains, but I think it was too high, I believe. Or couldn’t fit on the railways or something, but I believe it was the wrong trains or the trains were not engineered for our railways, whatever, something was wrong, I believe it was too high, I’m not sure. […] So, nothing happened, still the same. The only thing that changed is that they made the tickets more expensive. (MT20: 8)

While commuters’ hopes and expectations illustrated how the proximal agentive pathway ought to function, many examples from the data also indicated that their lived experience differed considerably. This helps to explain why the dimension of action potential is unconnected to this pathway. The breakdown in the function of this agentive pathway emphasized the challenges inherent in the mobility system.

#### The Collective Agentive Pathway

In relation to the challenges intrinsic to the Metrorail system, collective agency was perhaps the most informative and successful of the agentive pathways. Given that it is maximally distant to cluster 1 (containing all socio-structural environmental constraints and negative situational circumstance associations of mobility) in Figure 2, we can assume that it was the most functional of the agentive pathways. A re-contextualizing analysis of this agentive pathway revealed why this is the case. Collective agency was most frequently associated with social networks that commuters activated. These “cliques” consisted of friends, colleagues, or religious circles formed by commuting together. Here an example:

*Interviewee*: And there are also these cliques that form on trains. So everyone knows when they get on that this is their group that they chat with until they get off.

*Interviewer*: Do you have a group?

*Interviewee*: Yes, we have a group that meets in the mornings and we have church services on the train. So we are a group that meets on the train in the mornings and then hold a nice church service until we reach Stellenbosch station, until we get off at our station. There are a lot of networking groups and social networking groups that have formed because of it. And as a group, we also make sure that we meet once a month and go out for something to eat. (MT1: 2)

The functionality of these groups linked to a supportive or protective role they played in the lives of commuters. Not only did they support social and cultural activities, but they also provided safety and comfort to commuters confronted with uncomfortable, unreliable, and potentially dangerous commuting environments. In this way, the collective pathway helped to construct a protective buffer between commuters and the uncomfortable or potentially hostile mobility environment. Here are some examples:

Nothing bad has happened to me personally because I always travel in a group. It is a lot better if you travel in a group. And that’s the other thing when you take the train a lot then you meet and get to know people. And then people know you travel at that time every day and then you can sit in the same carriage and then you develop relationships like this. I have a group that I take the train with every night. And like when someone isn’t there you would message them and say “Where are you?” or “Are you late?” and so on. (MT4: 4)

So there is usually the thing that if I travel on a train then I need to be in a large group, large group being five or more people, safety in numbers. (MT27: 4)

I also once, it happened in the morning. I was writing [exams] that morning. So the trains were delayed and there had been delays from early in the morning. I had no other option ‘cause it was internal exams. With internal exams my teacher shows no mercy. If you’re late, you’re late, you’re not gonna write. And it was June so I needed the marks to apply to University. People started [she claps her hands loudly], the train came and people started to get on. I tried to get on, I tried, I fought and I fought. Then I could, one foot was on but the other foot was not. My bag was outside, my face was inside. I was holding on by the doors there, you know, onto the frame. I was holding by the door frame, so when the train was about to approach Bellville, it makes a turn but like a huge turn. I almost fell. If it was not for the person that was next to me, but a bit to the inside, I would have fallen. ‘Cause this guy saved my life, he just grabbed me by my shirt and tie and held onto me. And then I couldn’t breathe because I have asthma. I had already given up, I was going to die. But he pulled me in and other people also noticed that I was fainting. There was, I don’t know what happened, I don’t know where the people went, there was space, they made space. I was able to lie down and then they gave me a space to breathe, but I almost died. (MT15: 8)

#### The Function of Agentive Pathways

As stated earlier, individual, proxy, and collective agentive pathways lie on an agentive continuum. We found that these pathways varied according to the socio-structural, environmental impositions commuters experienced. Individual agentive pathways, for example, allowed individuals to respond to environmental constraints by selecting different mobility options to overcome or avoid problems and therewith created viable alternatives. Another strategy involved activating the proxy agentive pathway, which aimed to secure the help of more powerful actors, such as Metrorail or the government, to improve the mobility environment and to overcome socio-structural environmental impositions on behalf of commuters. While both strategies should have theoretically enabled someone to address, overcome, or avoid environmental constraints, few commuters were able to effectively implement personal agentive strategies to avoid Metrorail and most attempts at proxy agency seemed to fail at least in the short run to activate Metrorail or the government to improve regular commutes. It is within this context that the function and relative success of collective agency became most apparent. While collective efforts may not have been able to change the environment – they cannot prevent trains from being late, or services from being disrupted, or *skollies* and *tsotsis* (loosely translated, gangsters or criminals) from boarding trains – through interdependent effort, they provided a protective buffer that enhanced the action potential of commuters. By constructing an environment that offered resource and information sharing, coping mechanisms, and strength in numbers, this collective effort often provided the most functional agentive pathway of Metrorail users during their mobility encounters.

## Discussion and Conclusion

The purpose of this article is three-fold: to explore empirically Albert Bandura’s dimensions of agency and environment using the model of triadic reciprocal causation, to examine mobility as agency among Metrorail users in the Western Cape from this theoretical perspective, and to explore ways in which conceptions of mobility need to integrate technical, socio-structural, and psychosocial components in order to offer a context- and culture-sensitive approach to sustainable mobility. In applying Bandura’s framework, we identify empirically all intra- and inter-personal, as well as psychosocial and socio-structural dimensions that are part of his theory. We find that Bandura’s agency concept serves as a suitable analytic framework to systematize commuting experiences and practices among our Metrorail users. Mapping patterns of reciprocal interaction between agency and environment dimensions to study the interdependence between agentive dimensions enables us to visualize how mobility as agency unfolds along distinct pathways relating to individual, proxy, and collective agency. Agentive pathways lie on a continuum as agency moves from the individual to the proxy, with the collective occupying a central position. Another way to understand these agentive pathways refers to the function they serve in relation to the types of mobility environments. While the individual agentive pathway is closest to the classical understanding of personal agency or self-efficacy, and therefore representative of mobility achieved, in the context of Metrorail users, it remains largely aspirational, given that few of our commuters have access to alternative mobility modes. Proxies, such as Metrorail and the government, are critical to creating and mediating the mobility environment, and their failure to do so contributes to the restrictions and frustrations associated with the mobility system. In the context of Metrorail commuters in the Western Cape, it is the protective buffer of collective agency that enables commuters to achieve most consistently mobility as agency.

With regard to our first objective – to expand the concept of personal agency beyond the confines of unidirectional modes of causation adopted in studies on personal agency and self-efficacy, our application of Bandura’s framework of reciprocal causation shows that mobility as agency is inherently psychosocial and functionally dependent on technical and socio-structural dimensions. While our study supports evidence for the one-dimensional, intra-personal individual agentive pathway conventionally pursued in studies on personal agency (Bores-Rangel et al., 1990; Church et al., 1992; Ryerson, 1994; Hackett and Byars, 1996; Bandura, 1997, 2004a; Langlois et al., 1999; Rogers et al., 1999; Gibson, 2004; Chapman-Novakofski and Karduck, 2005; Hill et al., 2009; Tougas et al., 2015), we also identify other agentive pathways. To our knowledge, this is the first empirical exploration of the model of triadic reciprocal causation as proposed by Albert Bandura, and our study provides evidence for a more nuanced understanding of agency as distinct and systematic patterns of reciprocal interactions. Although our study is limited to a specific context – Metrorail commuters in the Western Cape, Bandura’s framework of triadic reciprocal causation and the mixed methods framework we adopt here serve as effective analytic tools to examine empirically this theory. Future research on agency and mobility in this vein ought to examine train systems and populations that differ in agency, environment, or region, or to study personal agency beyond mobility contexts to systematize how agentive pathways function more generally.

In contrast to some of Bandura’s critics, we find that the relative looseness of his concepts and their interdependence (Tschannen-Moran and Hoy, 2001; Garvis and Pendergast, 2016) prove to be an advantage. It enables us to use the model of triadic reciprocal causation to examine the interdependence between commuters and their environment without imposing *a priori* relationships. While Bandura defined the three types of agency and three types of environments, he did not define or operationalize how they are connected, arguing that this would vary according to context, culture, and other behavioral predispositions. Consequently, we could use the experiences of Metrorail commuters to identify how they and their environment shape mobility as agency, and how this functions in the context of Metrorail in the Western Cape. Given that agency is mediated by a constellation of contextual and cultural influences, the variability embedded in Bandura’s model provides an excellent framework to study personal agency in different settings, something future studies should pursue further.

The situated application of the model of triadic reciprocal causation expands what sustainable mobility may mean in a specific mobility context. To date, most studies in the mobility domain are limited to either intra-individual or structural concerns (Bergman et al., 2014; Bergman and Bergman, 2015; Cass and Faulconbridge, 2016), focusing on either infrastructure (Novaco, 2001; Brög et al., 2004; Hunecke et al., 2007) or commuter preference and behavior (Novaco, 2001; Steg and Vlek, 2009). When we examine the links between intra- and inter-personal, as well as socio-structural environmental dimensions of the Metrorail commuters we interviewed, our study concurs with Shepherd and Marshall (2005) findings that mobility practices are nested within an interdependent network of individual, social, and environmental factors. The reciprocal interactions between these dimensions have consequences on the day-to-day practices of commuters, and they highlight the weakness of policy approaches that fail to take this into account (see also Charlton, 2004; Steg and Gifford, 2005). Our study makes an empirical contribution toward systematizing the distinct patterns of reciprocal interaction between preferences and behaviors in conjunction with a specific context of a mobility environment. Based on our analysis of Metrorail commuters, we suspect that sustainable mobility policies aimed solely at individual behavior change or environmental and structural barriers are likely to have only limited success because the Metrorail environment is insufficiently aligned with different types of agency. They tend to lack the necessary attributes to enable a positive action potential, and in their current state, they do not connect sufficiently with the context and culture of commuters. Accordingly, we can make two policy recommendations: Individual commuter preferences and behaviors need to be conceptualized and understood in relation to a specific context and culture of mobility environments when formulating mobility solutions. And mobility interventions need to carefully blend hard and soft policy approaches while considering agency in a specific environment. Whereas most mobility approaches rightfully stress the importance of safe, reliable, and affordable public transport, they neglect what these three characteristics mean *in situ*, for example for employed or unemployed women living in townships or informal settlements. A mobility system that integrates contextual and cultural sensitivities would present a formidable baseline for agency beyond transportation. Our study invites policy makers to think in more complex ways about mobility systems. For example, in the South African context, the low prestige of train travel in relation to the high status of car ownership, particularly for males, needs to be considered when developing mobility solutions that integrate technological as well as motivational and emotional components.

The agentive pathways we studied here reflect the constellation of psychosocial and socio-structural environmental dimensions, which make up the mobility context of Metrorail commuters in the Western Cape. This context is characterized by extreme environmental constraints: overcrowded, dilapidated, outdated, and often unsafe trains and train infrastructure. Perhaps the most significant limitation to our study is its small scale and its specific Western Cape context, and future studies with a larger sample size could examine in more detail structural, contextual, and individual differences. While our study is thus not generalizable to a research group or geographic region, it nevertheless reveals how a theoretical framework serves well to illustrate different types of agency and their association with different types of mobility environments. Often, the ineffectiveness of a policy approach is best understood by transposing general policy assumptions into a specific context. Thus, in addition to this accomplishment, our study presented a thick description (Geertz, 1973) of everyday experiences of commuters in the Western Cape along a sophisticated psychological framework. Finally, our study highlights a promising approach for improving sustainable mobility systems beyond hard or soft policies. Removing obstacles that prevent agentive practices and taking into consideration different types of environments represent important steps toward developing context-specific and culture-sensitive sustainable mobility strategies.

## Author Contributions

The authors contributed equally to the theoretical and empirical work. ZB is the lead author.

### Conflict of Interest Statement

The authors declare that the research was conducted in the absence of any commercial or financial relationships that could be construed as a potential conflict of interest.

The reviewer NB and handling editor declared their shared affiliation at the time of the review.

## Footnotes

^1^In South Africa, the term ‘colored’ is colloquially used to denote people from a mixed race background.

## Abbreviations

CCA: content configuration analysis
HCA: hermeneutic content analysis
MDS: multidimensional scaling
SCT: social cognitive theory.
